# Water loss after stomatal closure: quantifying leaf minimum conductance and minimal water use in nine temperate European tree species during a severe drought

**DOI:** 10.1093/treephys/tpae027

**Published:** 2024-02-27

**Authors:** Songwei Wang, Günter Hoch, Georges Grun, Ansgar Kahmen

**Affiliations:** Department of Environmental Sciences – Botany, University of Basel, Schönbeinstrasse 6, 4056 Basel, Switzerland; Department of Environmental Sciences – Botany, University of Basel, Schönbeinstrasse 6, 4056 Basel, Switzerland; Department of Environmental Sciences – Botany, University of Basel, Schönbeinstrasse 6, 4056 Basel, Switzerland; Department of Environmental Sciences – Botany, University of Basel, Schönbeinstrasse 6, 4056 Basel, Switzerland

**Keywords:** canopy desiccation, drought tolerance, functional traits, hot temperature, minimum transpiration

## Abstract

Residual canopy transpiration (*E_min___canop_*) is a key physiological trait that determines trees’ survival time under drought after stomatal closure and after trees have limited access to soil water. *E_min___canop_* mainly depends on leaf minimum conductance (*g_min_*) and vapor pressure deficit. Here we determined the seasonal variation of *g_min_* and how *g_min_* is related to interspecies variation in leaf cuticular and stomatal traits for nine European tree species in a mature forest. In addition, we determined the species-specific temperature responses of *g_min_*. With this newly obtained insight, we calculated *E_min___canop_* for the nine species for one day at our research site during the 2022 central European hot drought. Our results show that at ambient temperatures *g_min_* ranged from 0.8 to 4.8 mmol m^−2^ s^−1^ across the nine species and was stable in most species throughout the growing season. The interspecies variation of *g_min_* was associated with leaf cuticular and stomatal traits. Additionally, *g_min_* exhibited strong temperature responses and increased, depending on species, by a factor of two to four in the range of 25–50 °C. For the studied species at the site, during a single hot drought day, *E_min___canop_* standardized by tree size (stem basal area) ranged from 2.0 to 36.7 L m^−2^, and non-standardized *E_min___canop_* for adult trees ranged from 0.3 to 5.3 L. *E_min___canop_* also exhibited species-specific rapid increases under hotter temperatures. Our results suggest that trees, depending on species, need reasonable amounts of water during a drought, even when stomates are fully closed. Species differences in *g_min_* and ultimately *E_min___canop_* can, together with other traits, affect the ability of a tree to keep its tissue hydrated during a drought and is likely to contribute to species-specific differences in drought vulnerability.

## Introduction

The intensity and frequency of both extreme high temperatures and droughts are predicted to increase worldwide in the 21st century (Teuling 2018). Already now, increasing drought-induced tree mortality has been reported across the globe (Allen et al. 2010, 2015, Rigling et al. 2013, Hammond et al. 2019, Powers et al. 2020, Schuldt et al. 2020). When it becomes difficult for trees to take up water from the soil, trees will close their stomata to maintain turgor pressure in their tissues and to avoid critical levels of dehydration and ultimately death (Bartlett et al. 2016, Martin-StPaul et al. 2017, Peters et al. 2023). Full stomatal closure cannot, however, completely prevent water loss from the canopy, and the transpiration through leaky stomata and (or) cuticle can lead to progressive dehydration of a tree (Schuster et al. 2017, Duursma et al. 2019). The dehydration rate and thus the surviving time of a tree after root water uptake is becoming limited in a severe drought will thus depend on the magnitude of residual canopy transpiration, the availability of stored mobile water inside the tree and the dehydration tolerance of critical tissues or organs (Blackman et al. 2016, Hammond and Adams 2019, Körner 2019, Martinez-Vilalta et al. 2019, Mantova et al. 2022).

Residual whole-tree canopy transpiration depends on leaf minimum water conductance (hereafter referred as *g_min_*), a tree’s total leaf surface area and the vapor pressure deficit (VPD) that the canopy experiences. However, the rates of residual whole-tree canopy transpiration during severe droughts and how these rates vary across species are rarely quantified. This is partly because the variability of *g_min_* is not well constrained. *g_min_* will vary across species, throughout a growing season as a consequence of leaf aging and in response to environmental drivers (Schuster et al. 2017, Duursma et al. 2019).

According to Schuster et al. (2017), for a total of 39 deciduous woody plants (trees and shrubs), *g_min_* ranges from 0.15 to 8.0 mmol m^−2^ s^−1^. Across-species variability in *g_min_* likely depends on cuticular and stomatal traits (Duursma et al. 2019, Machado et al. 2021). For example, a thicker leaf cuticle of *Arabidopsis thaliana* has been suggested to be an effective way to improve the plant’s drought performance, because of the accompanied lower *g_min_* (Kosma et al. 2009, Patwari et al. 2019). However, an effect of cuticular thickness on *g_min_* was not found in a range of different tree species, indicating that uncertainties remain regarding the morphological and anatomical leaf properties that determine *g_min_* (Bueno et al. 2020, Machado et al. 2021, Grünhofer et al. 2022). Additionally, Machado et al. (2021) reported that higher *g_min_* was associated with small and numerous stomata among 30 tropical savanna tree species and that most deciduous tree species had higher *g_min_* than evergreen trees.

As leaves mature and age, *g_min_* might vary not only among species but also within a growing season. Stable isotope studies have demonstrated that leaf waxes were synthesized mostly in the early stage of the leaf development (Kahmen et al. 2011, Gamarra and Kahmen 2016), and that cuticular water permeability was maintained throughout the lifespan of a leaf (Hauke and Schreiber 1998). However, physical damage on the leaf surface due to impacts of radiation, wind and rain erosion throughout the lifespan of a leaf can cause *g_min_* to vary seasonally with leaf aging, especially in evergreen conifer trees (Hadley and Smith 1994, Anfodillo et al. 2002, Jordan and Brodribb 2007). For instance, in the northeast Italian Alps, Anfodillo et al. (2002) reported that *g_min_* of *Picea abies* Karst. varied largely with the progression of the growing season in current-year needles, and increased fourfold from 5-month-old needles to 13-month-old needles. For the sake of simplicity, however, in most literature *g_min_* is generally considered a seasonally stable leaf trait, and potential effects of cuticle aging are not accounted for (Duursma et al. 2019).

Recently, several studies have reported a strong temperature sensitivity of *g_min_*, suggesting that *g_min_* will increase with rising leaf surface temperature (Eamus et al. 2008, Schuster et al. 2016, Bueno et al. 2019, Billon et al. 2020, Slot et al. 2021). For instance, Billon et al. (2020) compared the response of *g_min_* with a temperature gradient of 30–55 °C in five temperate tree species and found that *g_min_* increased nonlinearly under hotter temperatures. A species-specific phase transition temperature was also suggested, beyond which *g_min_* showed a rapid increase for some species (Bueno et al. 2019, Billon et al. 2020, Slot et al. 2021, Hartill et al. 2023). Given the anticipated more frequent hot temperatures in summer (Teuling 2018), the residual whole-tree canopy transpiration at full stomatal closure may thus be underestimated due to the temperature dependency of *g_min_*, and the surviving time of trees may be largely overestimated during such events (Billon et al. 2020, Breshears et al. 2021, Cochard 2021).

In this study, we conducted a comprehensive investigation of the variability of *g_min_* in nine ecologically and economically important temperate European tree species, and we used these data to calculate the residual whole-tree canopy transpiration at full stomatal closure (*E_min___canop_*) for these species using a 2022 hot drought event as a case study. Specifically, we studied for all species: (i) the intraspecific variability of *g_min_* across the growing season; (ii) the relationships between *g_min_* and leaf cuticular and stomatal traits; (iii) the temperature response of *g_min_*; and (iv) the *E_min___canop_* during a single day of the 2022 central European hot drought event.

## Materials and methods

### Research site and climate

This study was conducted with leaves collected from tree canopies at the Swiss Canopy Crane II (SCCII) research site (47°26′17″N, 7°46′37″E) close to Hölstein, about 20 km southeast of the city of Basel, Switzerland. The site is located on a plateau of the Jura Mountains at an elevation of 550 m a.s.l., and has high-clay soil and deep groundwater. *Fagus sylvatica* L. and *Picea abies* Karst. are dominant species at the site, forming a mixed temperate forest with 12 other tree species. At the center of the site is a 50-m tall canopy crane with a radius of 62.5 m that allows access to 333 trees.

The mean annual temperature and the mean annual precipitation in the region is 9.6 °C and 972 mm, respectively (mean data 1991–2020 from SwissMeteo, station Rünenberg). In 2022, the mean annual temperature and the total annual precipitation was 11.4 °C and 662 mm at the site. July was the hottest month with average and maximum temperatures of 21.0 and 34.8 °C, respectively, and a total precipitation of only 20 mm.

The nine species we investigated were *Acer pseudoplatanus* L., *Carpinus betulus* L., *Fagus sylvatica* L., *Fraxinus excelsior* L., *Quercus* sp. (hybrid forms by *Q. petrea* Liebl. and *Q. robur* L.), *Sorbus torminalis* Crantz, *Abies alba* Mill., *Picea abies* Karst. and *Pinus sylvestris* L. For simplicity, all tree species are referred by their genus names in the following.

### *g*_*min*_ measurements

Since vapor loss after stomatal closure is too low to be reliably detected by conventional gas exchange measurement methods, the approach of mass loss of detached leaves was used to determine *g_min_* (Duursma et al. 2019).

#### Species differences and seasonal variation of g_min_

Species differences and the seasonal variability of *g_min_* were investigated monthly from June to September 2020. Depending on the availability of trees under the canopy crane, two to five individual trees of each species were sampled (Table 1). Each month, two shoots (ca. 25 cm long) from two different branches were cut in the morning at the south-exposed side of the upper canopy of a tree using the canopy crane. The cut ends of the shoots were immediately submerged in water and transported in a cool box to the laboratory in Basel. In the lab, two healthy and fully expanded leaves from each shoot were used for *g_min_* measurements. Thus, four leaves were measured to calculate an average *g_min_* value for each tree and monthly averages of species were reported (i.e., *n* = 2–5). In *Fraxinus*, we used individual leaflets of the dissected leaf. For the three conifer species, the current-year and 1-year-old needles were measured separately using four fascicles in *Pinus* and four small twigs in *Abies* and *Picea*.

**Table 1:** *g_min_* and *g_max_* (mmol m^−2^ s^−1^) ± standard errors (SE) for nine studied species (*Acer pseudoplatanus, Carpinus betulus, Fagus sylvatica, Fraxinus excelsior, Quercus* sp., *Sorbus torminalis, Abies alba, Picea abies, Pinus sylvestris*).

Species	*g_min_* (mmol m^−2^ s^−1^)	Replicates (tree)	*g_max_* (mmol m^−2^ s^−1^)	Replicates (tree)
*Acer*	4.8 ± 0.4 a	5	275.7 ± 20.4 ab	4
*Carpinus*	1.2 ± 0.1 bd	5	149.6 ± 11.9 bcd	4
*Fagus*	2.5 ± 0.2 c	5	178.7 ± 28.7 abcd	4
*Fraxinus*	2.9 ± 0.4 ac	3	225.2 ± 6.5 abc	3
*Quercus*	2.7 ± 0.1 c	5	373.4 ± 58.0 a	4
*Sorbus*	4.8	2	369.7	2
*Abies*	1.5 ± 0.2 b	4	114.6 ± 7.2 d	4
*Picea*	1.5 ± 0.1 b	5	146.7 ± 30.3 cd	4
*Pinus*	0.8 ± 0.1 d	5	158.1 ± 18.4 bcd	4
Angiosperm	3.2 ± 0.6 A	–	262.1 ± 38.8 A	–
Gymnosperm	1.3 ± 0.2 A	–	139.8 ± 13.0 A	–

*g_min_* was measured at 20 °C during 2020 growing season. And *g_max_* was referred to *in situ* records of the canopy gas exchange during the growing seasons from 2020 to 2022 (unpublished data). Significant differences (*P* < 0.05) across species (functional groups) are indicated by lowercase (uppercase) letters.

For the measurements, leaves were cut at the petioles underwater with sharp razor blades from the shoot. Leaves, fascicles, or twigs were then rehydrated in de-gassed water via the standing rehydration method for 20 h in the dark (Arndt et al. 2015). After rehydration, the cut ends were sealed with high-melting paraffin wax (melting point 68 °C), and the saturated fresh weight of the leaves was immediately measured (*W_sat_*). All leaves were then placed into climate-controlled growth chambers to dry down for 30 h in the dark, with air temperature and relative humidity being stable at 20 °C and 69%, respectively. During the desiccation, leaves were taken out of the chambers at regular intervals (*ΔT*, 2–3 h) and weighed with a high precision balance (precision: ± 0.2 mg, Balance XPR204S, METTLER TOLEDO, Switzerland). This process took ~10 min. Leaf water loss (*W_loss_*) was determined based on the difference in leaf fresh weight (*W_fre_*) between two weighing intervals. After 30 h, leaves were removed from the growth chamber and weighed for the last time, and then completely dried in a drying oven for 72 h at 80 °C to obtain the leaf dry mass (*W_dry_*).

Leaf water conductance (*J,* mmol m^−2^ s^−1^) was calculated as


(1) \begin{equation*} J=\frac{W_{loss}}{\Delta T\ast A\ast VPD}\ast 98.0 \end{equation*}

where *A* is the total two-sided leaf area (m^2^). Leaf area was calculated based on values for specific leaf area (SLA) and dry mass of leaf samples after *g_min_* measurements. The SLA was calculated as the ratio of fresh leaf area to leaf dry mass. For this, leaves from adjacent twigs were scanned and fresh leaf area was extracted by our self-developed leaf image analysis tool (https://github.com/dabasler/LeafAreaExtraction). Leaves were then dried in the oven for 72 h to obtain dry mass. The bark area in conifer twigs was not considered in the calculation because of its rather small surface area compared with the attached total needle area. The VPD in Eq. (1) is the vapor pressure deficit (kPa), and the atmospheric pressure in the growth chamber was assumed to be 98.0 kPa. Additionally, the effect of boundary layer conductance on *g_min_* was assumed to be insignificant, since the fan system fully operated in the growth chamber during leaf desiccation. *g_min_* was obtained by plotting leaf water conductance over the remaining leaf relative water content (RWC; see Figure S1 available as Supplementary data at *Tree Physiology* Online). According to our preliminary experiment, full stomatal closure occurred at RWC < 80% (commonly after 2–3 h of exposure) for the studied tree species, and we assume that the leaf is functionally intact at RWC > 50%. At greater (including fatal) water deficits, there is a risk of artifacts because stomata may be pulled open by shrinking epidermis or cracks in the cuticle. Therefore, we used the horizontal part (values) of the curve between 80 and 50% of RWC to determine *g_min_* (Figure S1 available as Supplementary data at *Tree Physiology* Online). Leaf RWC was calculated as


(2) \begin{equation*} RWC=\frac{W_{fre}-{W}_{dry}}{W_{sat}-{W}_{dry}} \end{equation*}

#### Temperature sensitivity of g_min_ across species

To determine the temperature sensitivity of *g_min_*, field sampling started in mid July 2021 after the full expansion of new leaves in all nine species, and ended in late August 2021. For each species, two to four individual trees were sampled according to the cover range of the crane. At the south-exposed side of the upper canopy of each tree, a small shoot from each of two different branches was cut in the morning and the cut ends were kept in water in a cool box before the transportation to Basel. In the lab, five to seven healthy leaves from each shoot were cut under water and rehydrated as described above. For the three conifers, three or four twigs (including the current-year and 1-year-old needles) from each shoot were rehydrated.

For the temperature response of *g_min_*, we employed an alternative, less labor-intensive *g_min_* detection method as described above using the ‘DroughtBox’, an efficient tool for semi-automated measurements of *g_min_* under controlled environments (Billon et al. 2020). Fully rehydrated leaves and (or) twigs were hung in the DroughtBox to dehydrate progressively while the weights of leaves were automatically measured at 1 min intervals (precision: ± 50 mg). Meanwhile, three fans were running to ensure good air circulation inside the box. The balance of the DroughtBox is not sensitive enough to record the weight loss of individual leaves with high precision. For this reason, we jointly measured the weight loss of several leaves (10 to 14 leaves for broadleaf trees and six to eight twigs in conifers, as described above). *g_min_* measurements were performed for different leaves at temperatures of 25, 35, 45 and 50 °C, and the corresponding relative humidity was 37, 56, 63 and 66%. Relative humidity was moderately higher at high temperatures to slow the rate of water loss and to increase the precision of the *g_min_* measurements. Although a previous study showed that extremely high air relative humidity (>70%) caused a significant increase in cuticular permeability, the absolute difference in permeability was small and the effect of increasing temperature on cuticular permeability was several orders of magnitude higher than that caused by the changes in humidity (Schreiber et al. 2001). We tested if *g_min_* was dependent on relative humidity before our measurements but found no such effect (see Figure S2 available as Supplementary data at *Tree Physiology* Online).

To obtain a more complete picture of the temperature responses of *g_min_* in these nine species, *g_min_* measurements were repeated for all tree species under different temperatures in the DroughtBox from July to August 2022. Sampled shoots were directly rehydrated as described above and used for *g_min_* measurements (instead of isolated leaves). For the sake of simplicity, the bark area of the shoots was not considered in the calculation. Temperatures inside the DroughtBox were 30, 37, 43 and 48 °C, and the corresponding relative humidity was 40, 36, 33 and 37%. The average values of species based on individual trees were reported.

A nonlinear temperature response of *g_min_* has been reported for some species and phase transition temperatures had been extracted from these nonlinear functions (Eamus et al. 2008, Schuster et al. 2016, Bueno et al. 2019, Billon et al. 2020, Slot et al. 2021). In addition, there was no significant effect of sampling approaches or VPD on the temperature responses of *g_min_* in our data (see Tables S2 and S3 available as Supplementary data at *Tree Physiology* Online). For the sake of simplicity, the *g_min_* response to temperature in this study was thus visualized by fitting exponential functions to the pooled data across both years. Phase transition temperatures were determined using segmented regression.

### Stomatal traits

The epidermal impression technique was used to investigate leaf stomatal traits for deciduous species (Machado et al. 2021), except for *Acer* due to sunken stomata in the epidermal layer. During the field campaign 2022, two healthy twigs were collected in the morning from the same branches used in the *g_min_* measurements and transported to the laboratory in a cool box. Then two fully expanded leaves were re-cut under water from each twig. On the detached leaves, a small amount of clear nail polish was applied to both adaxial and abaxial leaf surfaces and allowed to dry for several minutes. After drying, nail polish was covered by transparent sticky tape and lifted. The tape with the leaf epidermis was trimmed and mounted onto microscopy slides. All slides were then observed with the aid of a light microscope (model CX43; Olympus, Tokyo, Japan), and photographed at ×400 magnification to calculate stomatal density (SD), and at ×1000 magnification for the measurements of stomatal size (SS). Picture analyses were done in ImageJ software (US National Institutes of Health). At least 10 pictures/slide (100–160 pictures in total for each species) and 15 clear stomata/slide (120–160 stomata in total for each species) were used to calculate SD and SS, respectively. The SS was determined as the guard cell length (L) multiplied by the width (W) of the guard cell pair (Franks et al. 2009). Additionally, the average fraction of the leaf epidermis that is allocated to stomata (*f_gc_*) was calculated by the average area of the guard cell pair and SD following de Boer et al. (2016)


(3) \begin{equation*} {f}_{gc}=\frac{\pi }{2}\ast W\ast L\ast SD \end{equation*}

Furthermore, the maximum stomatal conductance (*g_max_*) was extracted from our *in situ* records of the canopy gas exchange, which was monthly measured with a LiCOR 6800 (LICOR, Lincoln, NE, USA) during the growing seasons from 2020 to 2022 (unpublished data). All stomatal and following cuticular traits in this study were calculated on individual trees and reported as averages of species.

### Cuticular thickness

The hand-sectioning of fresh leaves was used to determine leaf cuticular thickness for six deciduous species. After the measurements of stomatal traits, one remaining leaf was cut from each twig (thus two leaves per tree). Each leaf was sectioned at the upper, middle and lower positions, respectively. All leaf sections were stained with Sudan III solution and then observed and photographed at ×400 magnification with the microscope described above. For each leaf section, five clear pictures were taken, and then the cuticular thickness was measured at different three clear points in each picture to obtain the mean values of different leaf cuticle positions. The measurements were conducted both on adaxial (CT_adaxial_) and abaxial cuticle (CT_abaxial_); subsequently, cuticular thickness (CT_total_) was calculated as the mean values of CT_adaxial_ and CT_abaxial_.

### The estimation of residual whole-tree canopy transpiration at full stomatal closure (*E*_*min*_*_*_*canop*_)

We calculated *E_min___canop_* for 3 August 2022, when the average predawn leaf water potential was about −2.0 MPa among nine species, and the minimum midday leaf water potential was close to the xylem water pressures inducing 12% loss of branch hydraulic conductivity (Ψ_12_) in most studied species (Arend et al. 2021, Kahmen et al. 2022, Peters et al. 2023). The diurnal records (from 8:00 a.m. to 8:00 p.m., time interval: 5 min) of canopy climate showed that maximum air temperature and lowest relative humidity were, respectively, 32.2 °C and 29.4%, and the highest VPD reached 3.4 kPa during that day (see Figure S3 available as Supplementary data at *Tree Physiology* Online). Although the dynamic changes in relative humidity were considerable during the day, the absolute humidity was rather stable (Figure S3 available as Supplementary data at *Tree Physiology* Online). So, this day represented a hot day during the 2022 summer drought that compares with the extreme days that trees had experienced in the 2015, 2018 and 2020 droughts in this area (Dietrich et al. 2018, Schuldt et al. 2020, Kahmen et al. 2022). We chose the diurnal patterns in air temperature and absolute humidity of this day to estimate *E_min___canop_* every 5 min (*ΔT*) from 8:00 a.m. to 8:00 p.m. for the nine investigated tree species and calculated the daily *E_min___canop_*.

To further simulate how temperatures increasing beyond the measured 32.2 °C on 3 August would impact *E_min___canop_*, we gradually elevated the original diurnal air temperature curve from 3 August by steps of 0.1 °C until a maximum temperature of 50 °C was reached (see Figure S4 available as Supplementary data at *Tree Physiology* Online). Meanwhile, we kept the corresponding daily absolute humidity curve unchanged so that VPD covaried with the raising temperature. Before our simulations, we noticed that leaf temperatures of the nine studied species were slightly higher than air temperatures during most of the daytime (unpublished data). However, the dynamic differences between leaf temperature and air temperature were highly species-dependent. For simplicity, we therefore assumed in our simulations that leaf temperatures were equal to air temperatures, but acknowledge that this assumption might lead to a slight underestimation of *E_min___canop_* for some species. Similarly, we also assumed in our calculation that leaf boundary layer conductance would not cause a significant impact on *E_min___canop_* for all species, which might also cause deviations in calculated results from *E_min___canop_* in nature.

Total canopy leaf area is an important variable for determining *E_min___canop_*. Total canopy leaf area is, however, highly variable among individual trees of a species and depends on tree size, stand density, growing conditions and forest management practices (Leuschner and Ellenberg 2017). For our calculations, we used the species’ measured projected leaf area for our research site (see Kahmen et al. 2022 for details). To standardize our data and make estimates of *E_min___canop_* of the given species comparable to other sites, we calculated the ratio of the average canopy projected leaf area to the average stem basal area for each species (*R*). Data for basal area per species were previously reported (Kahmen et al. 2022). As such, we calculated *E_min___canop_* as the daily residual whole-tree canopy transpiration at full stomatal closure per square meter of basal area following Eq. (4). In a second approach, we simply calculated *E_min___canop_* as a function of canopy leaf area ranging from 10 to 200 m^2^ for each species to estimate the actual daily water loss from varying canopy sizes in nature.


(4) \begin{equation*} {E}_{\mathit{\min}\_ canop}=\left({g}_{min}\ast VPD\right)/94.9\ast \Delta T\ast R \end{equation*}

where 94.9 (kPa) is air pressure at our research site. *ΔT* (s) is the time interval, and *R* is the ratio of the average canopy projected leaf area to the average stem basal area for each species (see Table 2).

**Table 2:** *E_min_canop_* (L m^−2^) and actual canopy water loss (L) for single trees of the nine studied species under three different maximum temperature conditions at the study site (*Acer pseudoplatanus*, *Carpinus betulus*, *Fagus sylvatica*, *Fraxinus excelsior*, *Quercus sp*., *Sorbus torminalis*, *Abies alba*, *Picea abies*, *Pinus sylvestris*).

		*E_min_canop_* (L m^−2^) standardized by basal area			*E_min_canop_* (L) for single trees at SCC II site		
	*T* (°C)	32.2	35	40	32.2	35	40
Species	*R*						
*Acer*	611.0	33.3	49.8	97.8	3.3	5.0	9.8
*Carpinus*	1046.6	24.6	34.9	62.1	1.5	2.1	3.7
*Fagus*	1161.3	36.7	50.5	85.6	2.2	3.0	5.1
*Fraxinus*	379.5	23.1	33.6	62.8	0.7	1	1.9
*Quercus*	401.9	21.1	30.7	57.5	5.3	7.7	14.4
*Sorbus*	406.3	30.9	45.0	84.2	2.8	4.1	7.6
*Abies*	274.2	6.9	10.6	21.9	0.3	0.4	0.9
*Picea*	836.8	13.8	19.0	32.3	1.0	1.3	2.3
*Pinus*	96.1	2.0	2.7	4.3	0.4	0.5	0.8

*T* indicates three different maximum temperature conditions in the simulation. *R* means the ratio of the average canopy projected leaf area to the average stem basal area for each species, data from Kahmen et al. (2022)

### Statistics

Seasonal significant differences in *g_min_* values were determined with the within-subjects one-way analysis of variance (ANOVA) in each species. Specifically, when hypothesis tests were met, one-way repeated measures ANOVA were used and followed by Student's T post hoc test. Otherwise, Friedman rank sum test was adopted in the analysis followed by Durbin–Conover post hoc test. At the same time, the significant differences between the two age groups of needles in each month were determined with Mann–Whitney *U* test. The significant differences in *g_min_* values across the nine species were determined based on the mean seasonal values of *g_min_* in each species. Additionally, considering that the especially high *g_min_* of the current-year needles in three conifer species only occurred at the early stage of the growing season, these special values were not taken into consideration in calculations. The results were analyzed with Kruskal–Wallis one-way ANOVA followed by Fisher LSD test, because homoscedasticity of variances was not fulfilled and the number of observations was relatively small among all species (*n* = 2–5). Then the significant difference in *g_min_* between the two functional groups was determined with Mann–Whitney *U* test because of the large difference in sample sizes between these two groups. Pearson linear correlation analyses were used to explore the relationships between *g_min_* and stomatal and cuticular traits among the species. Before analysis, species traits were log10-transformed, if necessary, to improve homoscedasticity and normality. It is worth noting that the values of *g_min_* at 25 °C and SLA both measured in 2021 were used in these Pearson linear correlation analyses. The significance threshold was 0.05 throughout all analyses. All the statistical analyses were performed in R v.4.1.2 (R Core Team, 2021).

## Results

### Seasonal course of *g*_*min*_

**Figure 1:**
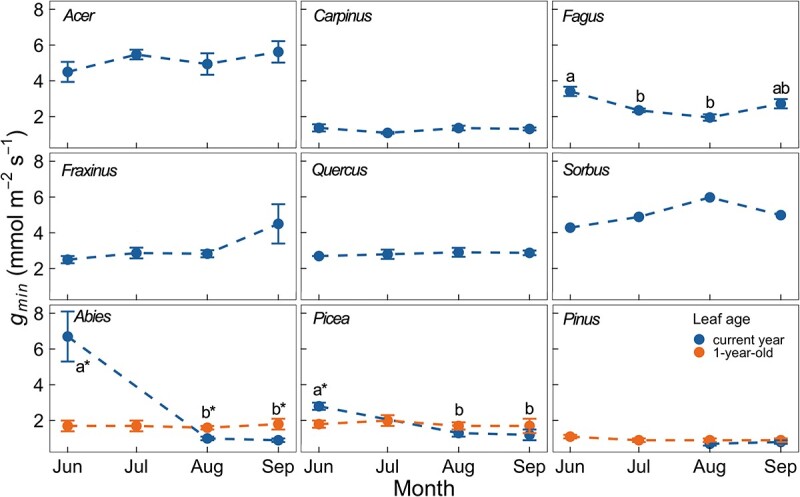
Seasonal variation of *g_min_* for nine species in 2020 measured at 20 °C. Different colours indicate the different leaf ages. Significant differences (*P* < 0.05) across months for current-year leaves within a single species are indicated by lowercase letters. Significant differences between the current and 1-year-old needles in each month are indicated by asterisks (^*^, *P* < 0.05). Each point is a mean value (*n* = 2–5), and the error bar represents the SE. Please note that in *Sorbus n* = 2, only the mean values are shown without SE.

Of the six deciduous broadleaf species, only *Fagus* showed significant monthly variation in *g_min_* in the 2020 growing season (*P* < 0.001, Figure 1). These variations were, however, small. The seasonal variations of *g_min_* in the other five species were small and not significantly different among sampling dates. For the three evergreen conifer species, where different needle generations were measured, *g_min_* was significantly higher at the first sampling date in June in current-year needles than in 1-year-old needles for *Abies* (*P* < 0.05) and *Picea* (*P* < 0.05), but this only occurred very early in the growing season (Figure 1). Then *g_min_* substantially decreased over the next month in the current year needles and there was no statistical difference between the two age groups of needles in *Picea* and *Pinus* after August. Additionally, we found that current-year needles exhibited significantly lower *g_min_* than 1-year-old needles for *Abies* in August and September (*P* < 0.05), but absolute differences were small. The 1-year-old needles showed non-significant seasonal variation in *g_min_* in all three conifer species.

### Species variation in *g*_*min*_

When averaged across the growing season of 2020, *g_min_* differed significantly among the nine species (*P* < 0.001, Table 1). *Acer* (4.8 ± 0.4 mmol m^−2^ s^−1^) and *Sorbus* (4.8 mmol m^−2^ s^−1^) had similar *g_min_* values and were the highest of the nine studied species. In deciduous broadleaf species, *Carpinus* (1.2 ± 0.1 mmol m^−2^ s^−1^) had the lowest *g_min_*. The lowest *g_min_* among all nine studied species was found in *Pinus* with only 0.8 ± 0.1 mmol m^−2^ s^−1^, which was also significantly lower than that in *Abies* (1.5 ± 0.2 mmol m^−2^ s^−1^) and *Picea* (1.5 ± 0.1 mmol m^−2^ s^−1^). While there was a tendency for *g_min_* being higher in angiosperms than in gymnosperm trees, this difference was not significant (*P =* 0.09). Compared with the *g_max_* of the nine studied species, *g_min_* was ~1–2% of *g_max_* in all species (Table 1).

### The relationship between *g*_*min*_ and stomatal and cuticular traits across species

**Figure 2:**
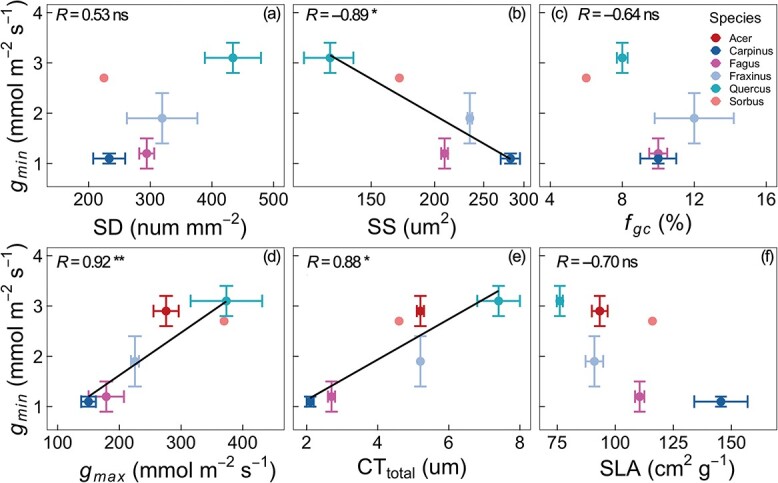
Relationships between *g_min_* measured in the 2021 growing season at 25 °C and stomatal density (SD) (a), stomatal size (SS) (b), the fraction of epidermis allocated to stomata (*f_gc_*) (c), the maximum stomatal conductance (*g_max_*) (d), total cuticular thickness (CT_total_) (e) and SLA (f). Each point represents the mean value for a species (*n* = 2–4), and the error bar represents the SE. The black lines indicate the fitted regression lines. Pearson correlation coefficients are shown; ns, nonsignificant; ^**^, *P* < 0.01; ^*^, *P* < 0.05.

*g_min_* measured at 25 °C in the 2021 growing season was strongly negatively correlated with SS (Figure 2b). Besides that, a strong positive correlation was also observed between *g_min_* and *g_max_* (Figure 2d). We found a significant difference in cuticular thickness between the adaxial (upper) and abaxial (lower) leaf sides, the former being twice as thick as the latter (see Figure S6 available as Supplementary data at *Tree Physiology* Online). *g_min_* was positively correlated with both CT_total_ and CT_adaxial_ (Figure 2e, Figure S5a available as Supplementary data at *Tree Physiology* Online), but not with CT_abaxial_ (Figure S5b available as Supplementary data at *Tree Physiology* Online). There was also a strong correlation between CT_total_ and SLA (Figure S5c available as Supplementary data at *Tree Physiology* Online), but there was no significant correlation between *g_min_* and SLA (Figure 2f). And there was no significant correlation between SD and SS (Figure S5d available as Supplementary data at *Tree Physiology* Online).

### The temperature response of *g*_*min*_

**Figure 3:**
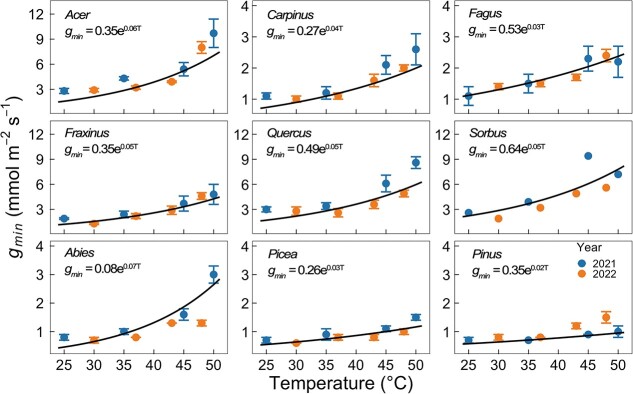
Temperature response of *g_min_* for nine species. Each point is the mean value (*n* = 2–4), and the error bar represents the SE. Different colors indicate the different sample years. The solid black lines and equations illustrate the significant exponential models for pooled data. Please note *n* = 2 in *Sorbus*, only the mean values are shown without SE.

With temperature increasing from 25 to 50 °C, *g_min_* measured in the 2021 and 2022 growing seasons increased across all studied species (Figure 3, also see Table S1 available as Supplementary data at *Tree Physiology* Online). In deciduous broadleaf species, the strongest temperature response of *g_min_* was observed in *Acer,* where *g_min_* increased by a factor of 3.5 from 25 to 50 °C (2.8 to 9.7 mmol m^−2^ s^−1^, Table S1 available as Supplementary data at *Tree Physiology* Online). By contrast, *Fagus* was the least responsive to increasing temperatures, with an approximate double increase in *g_min_.* In conifer species, *Abies* showed a stronger temperature response than *Picea* and *Pinus*, with its *g_min_* increasing by a factor of 3.8 from 25 to 50 °C (0.8 to 3.0 mmol m^−2^ s^−1^, Table S1 available as Supplementary data at *Tree Physiology* Online). By applying segmented linear regression, significant phase transition temperatures were determined only in *Acer* (Table S1 available as Supplementary data at *Tree Physiology* Online, 43.4 ± 0.9 °C, *P* < 0.001), *Carpinus* (38.4 ± 3.1 °C, *P* < 0.001) and *Fraxinus* (40.6 ± 2.7 °C, *P* < 0.001).

### Residual whole-tree canopy transpiration at full stomatal closure (*E*_*min*_*_*_*canop*_)

**Figure 4:**
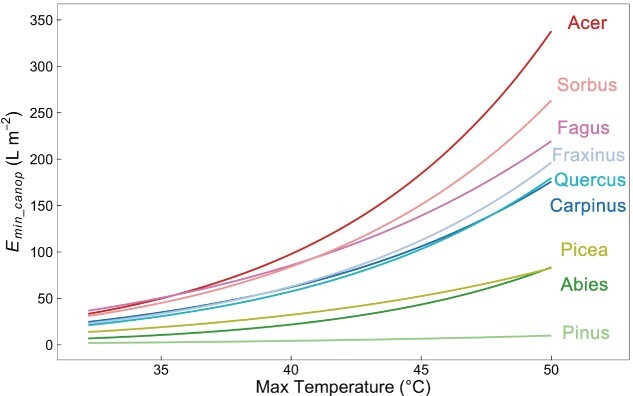
Estimation of residual whole-tree canopy transpiration at full stomatal closure (*E_min___canop_*) at different air temperatures standardized to tree size by stem basal area for nine tree species during a day of the 2022 summer drought.

Using environmental data that were recorded during a summer drought in 2022, we determined the residual whole-tree canopy transpiration at full stomatal closure during a single hot day by calculating the amount of water transpired by a tree canopy in terms of square meters of stem basal area. Under the natural temperature condition, namely a maximum temperature of 32.2 °C, *Fagus* had the highest *E_min___canop_* of 36.7 L m^−2^, followed by *Acer* (33.3 L m^−2^) and *Sorbus* (30.9 L m^−2^) (Figure 4, Table 2). The lowest *E_min___canop_* was observed in *Pinus* with about 2.0 L m^−2^. As we increased the temperature in our simulation, these nine species showed varying degrees of increase in *E_min___canop_*. After the maximum temperature was beyond 35.5 °C, *E_min___canop_* of *Acer* became higher than that of *Fagus* (Figure 4). Similarly, *Fraxinus* showed higher *E_min___canop_* than *Carpinus* when the maximum temperature was above 39.0 °C. Compared with other species, *Acer* and *Abies* exhibited the strongest temperature responses of *E_min___canop_* when the maximum temperature rose from 32.2 to 40.0 °C, with an almost threefold increase (Table 2).

**Figure 5:**
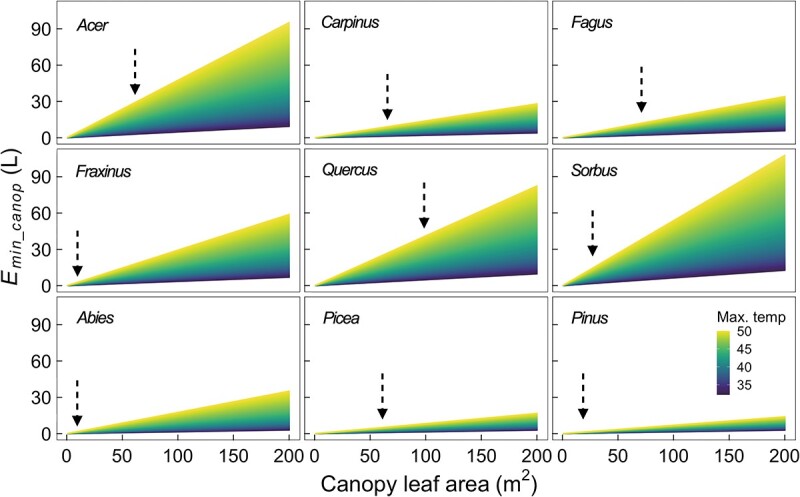
Non-standardized estimation of daily water use at full stomatal closure (*E_min___canop_*) for nine tree species as a function of canopy leaf areas and temperature settings during a day of the 2022 summer drought. The black arrows indicate the average canopy leaf area for a single adult tree at the Swiss Canopy Crane II (SCCII) research site.

The non-standardized amount of canopy transpiration at 32.2 °C and full stomatal closure for an adult tree during a single hot drought day was the highest in *Quercus* (Table 2), about 5.3 L at our research site, followed by *Acer* (3.3 L) and *Sorbus* (2.8 L). By contrast, *Abies* only lost 0.3 L, which was lower than *Pinus* (0.4 L) and *Picea* (1.0 L). Additionally, canopy water loss increased two to three times for all species, when the maximum temperature increased from 32.2 to 40.0 °C (Table 2, Figure 5).

## Discussion

This study shows that *g_min_* measured at ambient temperatures of 20 °C varied considerably among the nine studied species, ranging from 0.8 to 4.8 mmol m^−2^ s^−1^. This large interspecies difference was associated with leaf cuticular and stomatal traits. Although seasonal variations of *g_min_* were observed in a few species, most species kept rather stable *g_min_* values throughout the growing season. *g_min_* exhibited strong temperature responses in all studied species and it generally increased by a factor of two to four in a temperature gradient of 25–50 °C. Based on our estimation, *E_min___canop_* standardized by stem basal area ranged from 2.0 to 36.7 L m^−2^ during a hot drought summer day with a maximum temperature of 32.2 °C and increased to 4.3–97.8 L m^−2^ when the simulated maximum temperature was 40 °C. At 32 °C, an adult tree at our research site transpired between 0.3 and 5.3 L day^−1^ which increased to 0.8 and 14.4 L day^−1^ at 40 °C. Regarding the risk of canopy water loss caused by high *E_min___canop_* under compound hot drought, *Acer* was the most vulnerable and *Pinus* the most resistant tree among the species that we investigated.

### Seasonal variation of *g*_*min*_

Knowledge of the seasonal variation of *g_min_* is scarce in the literature. Hauke and Schreiber (1998) found that *Hedera helix* had a constant cuticular conductance throughout the growing season after the first 30 days of leaf development. On the contrary, Hadley and Smith (1994) observed that *g_min_* of *Picea engelmannii* increased by 40% in the current-year needles after the first winter in the Central Rocky Mountains, USA, and Heinsoo and Koppel (1998) found a rapid decline of *g_min_* in *Picea abies* in the first weeks after budbreak.

There is multiple evidence that the leaf cuticle typically forms early during leaf ontogeny and then keeps a constant water permeability during the rest of the season (Hauke and Schreiber 1998, Sachse et al. 2010, Kahmen et al. 2011, Gamarra and Kahmen 2016). At our research site, all deciduous tree species fully unfolded their leaves in early May 2020 (Zahnd et al. 2023). Our fieldwork started in the middle of June when leaves were already fully developed. Although we observed statistically significant monthly variations of *g_min_* in *Fagus* (Figure 1), these fine-scale fluctuations were unlikely to have profound effects on canopy water loss. Accordingly, we conclude that after leaf maturation, most deciduous tree species have a stable *g_min_* throughout the growing season.

Spring bud break of the three evergreen conifer species commenced in late April (*Abies* and *Picea*) and in late May (*Pinus*) 2020 (Zahnd et al. 2023). We observed a rapid decline in *g_min_* of the current-year needles early in the growing season and found significant differences in *g_min_* in different needle-age groups, with older needles having a slightly higher *g_min_* than fully matured current-year needles. Similarly, Heinsoo and Koppel (1998) observed a fast decline of *g_min_* in Norway spruce during the first weeks of growth, followed by a gradual increase in *g_min_* with needle age (up to 5 years). Although, in this study, the differences of <1 mmol m^−2^ s^−1^ in *g_min_* between the current-year and 1-year-old needles will only make a physiologically insignificant difference for canopy water loss at full stomatal closure, we think the potential changes of *g_min_* with leaf age should be kept in mind when considering the drought vulnerability of evergreen tree species.

### Interspecies variation of *g*_*min*_ and its association with leaf morphology and anatomy

The *g_min_* values of the nine temperate European tree species reported here are within the range of previously published values for other species (see data review of Schuster et al. 2017, Duursma et al. 2019). Our data therefore confirm that *g_min_* is about two orders of magnitude smaller than the maximum stomatal conductance of trees and ~1–2% of *g_max_* of the species at our study site (Figure 2d, Table 1). At the same time, we observed a large variability in *g_min_* among the nine studied species. The highest *g_min_* values were detected for *Acer* and *Sorbus* with about 4.8 mmol m^−2^ s^−1^, which were more than six times higher than the lowest *g_min_* observed for *Pinus*. We also found a trend that gymnosperm trees had a generally lower *g_min_* than angiosperm species, but we found no significant difference between these two functional groups. This result is in concert with the findings of Schuster et al. (2017), who showed no significant difference in *g_min_* among 11 life form groups, including deciduous trees and evergreen conifers. In this study, *g_min_* was very low in some deciduous species (e.g., *Carpinus,* 1.2 mmol m^−2^ s^−1^), similar to that of conifers. By contrast, in a total of 54 tropical tree species, Machado et al. (2021) and Slot et al. (2021) found that *g_min_* was higher in deciduous than in evergreen tree species.

We found a strong positive correlation between *g_min_* and CT_total_ (Figure 2e). At first glance, this may come as a surprise as it is very common to hypothesize that thicker cuticle can reduce water permeability, and thus lead to lower *g_min_*. Yet, different lines of evidence suggest a weak role of cuticular thickness in the control of cuticular water permeability (Riederer and Schreiber 2001, Bueno et al. 2020, Machado et al. 2021). Rather, the leakiness of the cuticle may rest on the integrity and chemical composition of the wax layer of the cuticle, instead of the wax amounts (Jetter and Riederer 2016, Grünhofer et al. 2022). In a recent study, Grünhofer et al. (2022) demonstrated that an up to 12.5-fold higher wax coverage was unable to reduce the residual water loss in detached leaves of *Populus* × *canescens*. Additionally, it is worth noting that in this study *g_min_* was correlated to CT_adaxial_ rather than CT_abaxial_ (Figure S5 available as Supplementary data at *Tree Physiology* Online), and that there was an average two-fold difference between CT_adaxial_ and CT_abaxial_ (Figure S6 available as Supplementary data at *Tree Physiology* Online). So, the positive correlation between *g_min_* and CT_total_ may imply the necessity of increasing wax amounts on leaf adaxial surface to enhance the defense of the leaf against harsh canopy microclimates. The negative correlation between CT_total_ and SLA also indicated this carbon investment in leaf construction (Figure S5c available as Supplementary data at *Tree Physiology* Online).

Previous studies have reported that higher *g_min_* values were associated with higher SD, *f_gc_* and smaller SS (Muchow and Sinclair 1989, Machado et al. 2021). In this study, we detected indeed a negative correlation between SS and *g_min_*, but no significant correlations between SD, *f_gc_* and *g_min_* (Figure 2). For the poor statistical performance in the latter, one of the potential explanations was due to the small number of investigated tree species and a large interspecies variation in stomatal morphology among these tree species. We also explored the correlation between gas exchange capacity and *g_min_* and found that *g_max_* was tightly and positively correlated with *g_min_*. This result was in agreement with those reported by Machado et al. (2021), in which *g_max_* was estimated by the theoretical link between stomatal conductance and stomatal morphological traits. It is therefore plausible that a trade-off exists, in which canopy leaves have a higher *g_min_* and bear higher dehydration risk while increasing the gas exchange rate.

### Temperature response of *g*_*min*_

Temperature response of *g_min_* has been shown for plant species across different lifeforms from contrasting climates, including tropical broadleaf tree species (Slot et al. 2021), temperate evergreen conifers (Billon et al. 2020), cool-temperate evergreen angiosperms (Hartill et al. 2023) and desert vine (Bueno et al. 2019). All temperate tree species that we tested in this study also showed a significant increase in *g_min_* with rising temperatures, as well as a large difference in the thermal sensitivities of *g_min_*. According to Schuster et al. (2016), the temperature response of *g_min_* can be possibly ascribed to the change in the structure of either the polymer chains in the cutin matrix or the crystalline wax barrier, resulting in increasing cuticular water permeability and thus higher *g_min_*. To a certain degree, this mechanism may shed light on the highly species-specific thermal sensitivities of *g_min_* that we found in the study. Additionally, it is worth noting that only sunlit leaves from the south-exposed side of the upper canopy were investigated in this study, considering that the structure of the forest canopy at the site is rather open, with a basal area of 24.6 m^2^ ha^−1^ and an average leaf area index of ca 2.2 (Zahnd et al. 2023). There are remaining uncertainties in the temperature response of *g_min_* for shade leaves at lower canopy positions, and they deserve closer scrutiny. For example, Slot et al. (2021) found that sun leaves generally had significantly higher *g_min_* than shade leaves for some tropical tree species, but there was no evident interaction between the temperature response of *g_min_* and the position of leaves in the canopy.

Although sampling in 2 years with different approaches had minor effects on the temperature responses of *g_min_* for the studied tree species, all data showed a strong exponential increase in *g_min_* with temperature. However, we cannot identify whether these differences are caused by the different methods we used or if these differences are because samples originated from different years and thus reflect year-to-year variability in *g_min_*. According to our study of *g_min_* in a provenance trial, both *g_min_* and its thermal sensitivity can exhibit strong phenotypic plasticity with changing hydroclimate in some tree species (unpublished data), which may hint toward year-to-year differences in *g_min_* as a reason for the observed variability.

Several studies also revealed a phase transition temperature in the thermal sensitivity of *g_min_*, suggesting dramatic changes in the structure of cuticular wax under certain hot temperatures (Schuster et al. 2016, Bueno et al. 2019, Billon et al. 2020, Slot et al. 2021, Hartill et al. 2023). This temperature threshold was determined by different approaches, such as by data transformation (Arrhenius plot, Billon et al. 2020) or directly using a segmented bi-linear function (Slot et al. 2021). For our nine studied tree species, similarly, phase transition temperatures could also be determined in three deciduous broadleaf tree species using the bi-linear function, ranging from 38.4 to 43.4 °C (see Table S1 available as Supplementary data at *Tree Physiology* Online). Ecologically, however, these different methods all yielded similar core knowledge of the thermal sensitivity of *g_min_*, namely the non-linear increase in *g_min_* under compound hot drought. In the study, temperature responses of *g_min_* all showed significantly exponential changes under hotter temperatures.

### Residual whole-tree canopy transpiration at full stomatal closure (*E*_*min*_*_*_*canop*_)

The time for which a tree can remain hydrated with complete stomatal closure after the dysfunction of the capillary continuum from soil to the root is strongly influenced by *E_min___canop_* (Blackman et al. 2019, Billon et al. 2020, Lemaire et al. 2021, Challis et al. 2022). We estimated *E_min___canop_* for the nine tree species at our research site for a hot day during the 2022 Central European hot drought. According to monthly predawn and midday canopy leaf water potential measurements during the growing season 2022 at the research site (unpublished data), all nine studied species experienced severe soil water shortage in July and August, at which time their stomates were typically closed (Dietrich et al. 2018, Peters et al. 2023). Based on our estimation, the maximum difference in *E_min___canop_* between nine studied tree species was about 18-fold either when standardized by stem basal area or considering the actual water loss of individual species for trees at our research site. *Fagus*, *Acer* and *Sorbus* had relatively high *E_min___canop_* values and they may thus encounter a higher risk of canopy desiccation than other species under severe droughts, once water supply from the soil has become extremely limited. However, there are significant differences in root water uptake depth among the nine tree species that we studied at our research site (Brinkmann et al. 2019, Kahmen et al. 2022), which might compensate for high values of *E_min___canop_* in some species, including *Acer*, *Quercus* and *Fraxinus*. Others, including *Fagus*, *Carpinus* and *Picea*, mainly rely on shallow root water uptake (Kahmen et al. 2022). In the peak of the 2022 summer hot drought (July and August), the mean minimum predawn branch water potentials (Ψ_min_) were − 1.98 ± 0.36 (mean ± SD, *n* = 12) and − 2.02 ± 0.3 (*n* = 7) MPa for *Quercus* and *Acer*, respectively (unpublished data). At the same time, these values were −2.49 ± 0.2 (*n* = 12) and −2.89 ± 0.42 (*n* = 13) MPa for *Picea* and *Fagus*. It is worth noting that the mean Ψ_min_ was lower than Ψ_12_ in *Fagus* (−2.74 ± 0.16 MPa). Thus, the water leakiness caused by high *E_min___canop_* in *Acer* and *Quercus* may be compensated by access to deep soil water during a drought. In contrast, the combination of shallow root water uptake depth and high *E_min___canop_*, as observed in *Fagus*, is likely to accelerate the risk of this species being severely impacted by drought as observed across Europe in recent years (Arend et al. 2022, Frei et al. 2022, Kahmen et al. 2022).

Under severe drought conditions, the demand of stem water flux for transpiration will dramatically decrease because of the full stomatal closure (Duursma et al. 2019, Körner 2019). At our research site, the water consumption of trees at full stomatal closure during a hot dry summer day can be as low as 0.3 L but may be as high as 5.3 L (Table 2), depending on the species. Importantly, our simulations demonstrate that hotter temperatures can lead to an exponential increase in *E_min___canop_* for all studied tree species, due to the simultaneous increase in VPD and *g_min_*. Our results suggest that the resistance to canopy water loss decreased to varying degrees with increasing temperature in different tree species. For instance, when the maximum temperature was below 35.5 °C, *Fagus* exhibited higher *E_min___canop_* than *Acer*, despite the higher *g_min_* of *Acer*, indicating the large contribution of more leaves associated with each square meter of the basal area to *E_min___canop_* in *Fagus* (Table 2). Beyond 35.5 °C, however, *Acer* showed a higher *E_min___canop_* than *Fagus*, which was caused by the stronger temperature response of *g_min_* in *Acer*. Similarly, *E_min___canop_* of *Fraxinus* exceeded that of *Carpinus* when the maximum temperature was beyond 39 °C, implying the changing risks of canopy desiccation among these nine tree species under hotter environments. Thus, with the increasing severity of compound hot droughts, interspecies drought vulnerabilities may become more elusive, as some tree species that are considered drought-tolerant (with lower *E_min___canop_*) in current climatic environments may become more vulnerable than other species.

Although standardizing *E_min___canop_* based on the ratio of canopy leaf area to the basal area in the study allowed us to compare *E_min___canop_* among the nine studied species, some caveats should be considered. Firstly, the ratio of canopy leaf area to basal area used in the standardization will have a strong influence on *E_min___canop_* and it might change in different forests due to differences in tree size, stand density, leaf area index, growing conditions and forest management practices (Leuschner and Ellenberg 2017). Secondly, there are few reports on the temperature response of *g_min_* in tree species, especially on the genetic variation and environmental plasticity of the thermal sensitivity of *g_min_* (Cochard 2021). For instance, the variation of *g_min_* across the canopy gradient, which we discussed earlier, may lead to biased estimates of *E_min___canop_* and influence the assessment of canopy desiccation vulnerability in different tree species, and thus warrant future investigations.

## Conclusion

This study demonstrates that *g_min_* varies considerably among nine studied species and is tightly associated with leaf cuticular and stomatal traits. The seasonal variation in *g_min_* was observed only in few species, and such slight changes are unlikely to cause profound influences on leaf water loss. Importantly, this study shows that *g_min_* responds to the instantaneous change in air temperature and increases strongly with rising air temperature. Ultimately, there were as large as 18 times differences in the *E_min___canop_* among these species during the peak of hot drought 2022 and the resistance of different tree species to *E_min___canop_* decreased at varying degrees under hotter temperatures. This study emphasizes the importance of *g_min_* and its temperature response as one of the dominant variables that are responsible for species differences in *E_min___canop_*. Our data show that even when stomates are fully closed during a hot drought, different tree species need substantial amounts of water to remain hydrated. The amounts of water needed vary among species and can, in combination with other traits (root water uptake depth, tissue desiccation tolerance and capacitance), contribute to understanding species differences in drought vulnerability.

## Supplementary Material

Supplementary_material_tpae027

## Data and availability statement

Data that support the findings of this study will be made available upon request.
