# Supplementary material for: Water loss after stomatal closure: quantifying leaf minimum conductance and minimal water use in nine temperate European tree species during a severe drought
Source: Tree Physiol. 2024 Feb 27;44(4):tpae027. doi: 10.1093/treephys/tpae027 (PMC10993720; doi:10.1093/treephys/tpae027)
Supplement: Supplementary_material_tpae027 [file supplementary_material_tpae027.docx]

**Supplementary Material to “Water loss after stomatal closure: Quantifying leaf minimum conductance and minimal water use in 9 temperate European tree species during a severe drought”**

**

**

**Fig. S1.** Leaf water conductance (Leaf_wc_) as a function of the relative water content for two species (a: *Quercus sp*., b: *Abies alba*). Each point represents an individual measurement at 20 °C. A smoothing curve based on generalized additive mode is fitted to aid the eye. Shade area indicates the occurrence of *g_min_* during dehydration process, and in which the mean of measured values was used for *g_min_*





**Fig. S2.** Comparison of leaf minimum conductance (*g_min_*) under 40 °C with different relative humidity conditions for 2 tree species (a: *Fagus sylvatica*, b: *Pinus sylvetris*). No significant humidity effect on *g_min_* was observed


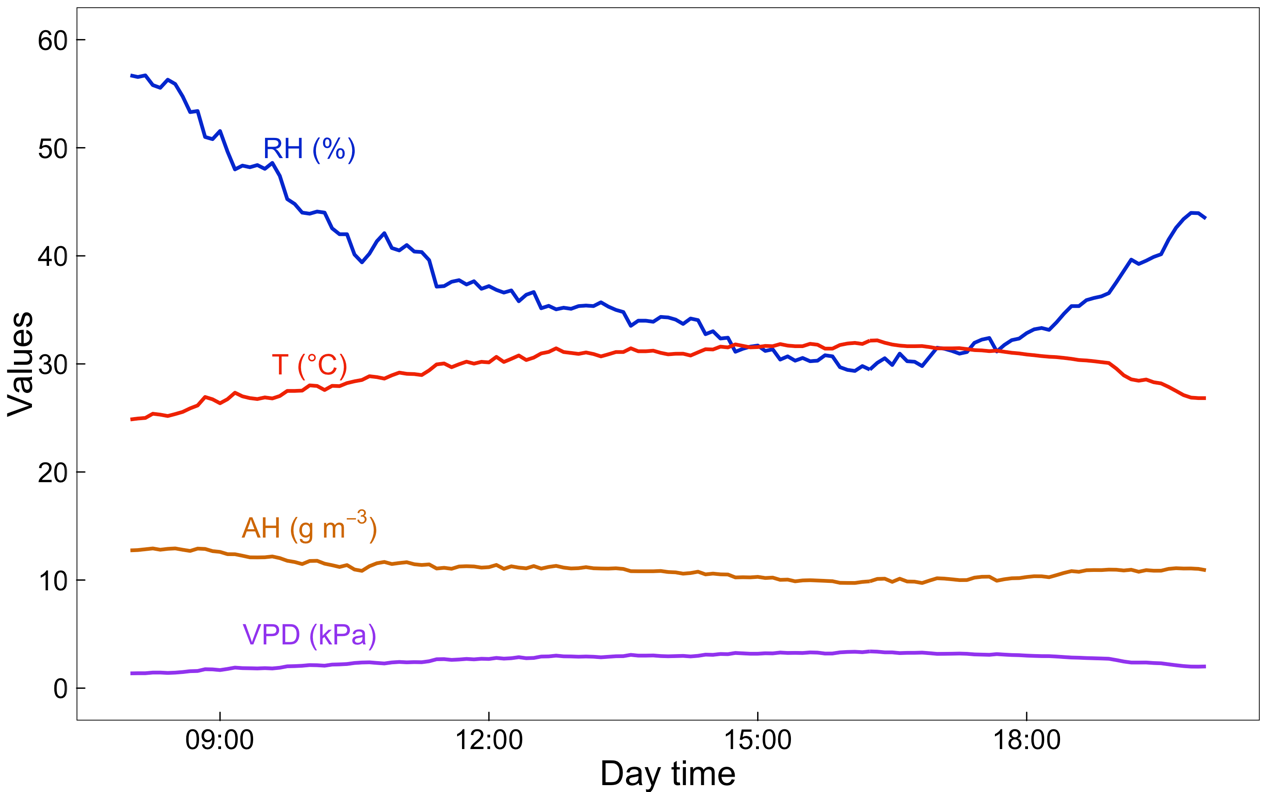


**Fig. S3.** The dynamic pattern of the canopy climate at the research site between 8:00 and 20:00 on 3^rd^ August 2022. Blue line is relative humidity (RH, %), red line is air temperature (T, °C), brown line is absolute humidity (AH, g m^-3^), purple line is vapor pressure deficit (VPD, kPa)





**Fig. S4.** The schema of the estimation of the canopy climate under hotter and drier circumstances. (a): based on the dynamic pattern of the canopy climate from 8:00 to 20:00 on 3^rd^ August 2022 (blue line), the temperature curve is elevated by 0.1 °C to that maximum of from 32.2 °C on 3^rd^ August to 50 °C. (b) vapor pressure deficit (VPD) is covarying with elevated air temperature





**Fig. S5.** Relationships between (a) leaf minimum conductance (*g_min_*) and adaxial cuticular thickness (CT_adaxial_), (b) leaf minimum conductance (*g_min_*) and abaxial cuticular thickness (CT_abaxial_), (c) specific leaf area (SLA) and total cuticular thickness (CT_total_), (d) stomatal density (SD) and stomatal size (SS). Each point represents mean value for each species (N = 2 – 4), and the error bar represents the SE. The black lines indicate the significant fitted regression line, and Pearson correlation coefficients are shown; ns, nonsignificant; *, *p* < 0.05





**Fig. S6.** Comparison of thickness between abaxial and adaxial cuticle for 6 deciduous broadleaf specie

**Table S1.** *g_min_* (mmol m^-2^ s^-1^) ± standard errors for nine studied species (*Acer pseudoplatanus*, *Carpinus betulus*, *Fagus sylvatica*, *Fraxinus excelsior*, *Quercus sp*., *Sorbus torminalis*, *Abies alba*, *Picea abies*, *Pinus sylvestris*) at the different temperature levels and phase transition temperature (*T_p_*) value for each specie

| Temperature (°C) | *g_min_* (mmol m^-2^ s^-1^) | | | | | | | | |
| --- | --- | --- | --- | --- | --- | --- | --- | --- | --- |
|  | *Acer* | *Carpinus* | *Fagus* | *Fraxinus* | *Quercus* | *Sorbus* | *Abies* | *Picea* | *Pinus* |
| 25 | 2.8 ± 0.3 | 1.1 ± 0.1 | 1.1 ± 0.3 | 1.9 ± 0.1 | 3.0 ± 0.3 | 2.6 | 0.8 ± 0.1 | 0.7 ± 0.1 | 0.7 ± 0.1 |
| 30 | 2.9 ± 0.2 | 1.0 ± 0.1 | 1.4 ± 0.1 | 1.3 ± 0.1 | 2.8 ± 0.5 | 1.9 | 0.7 ± 0.1 | 0.6 ± 0.0 | 0.8 ± 0.1 |
| 35 | 4.3 ± 0.2 | 1.2 ± 0.2 | 1.5 ± 0.3 | 2.4 ± 0.4 | 3.4 ± 0.4 | 3.9 | 1.0 ± 0.1 | 0.9 ± 0.2 | 0.7 ± 0.0 |
| 37 | 3.2 ± 0.2 | 1.1 ± 0.1 | 1.5 ± 0.1 | 2.2 ± 0.3 | 2.6 ± 0.5 | 3.2 | 0.8 ± 0.0 | 0.8 ± 0.1 | 0.8 ± 0.0 |
| 43 | 3.9 ± 0.1 | 1.6 ± 0.2 | 1.7 ± 0.1 | 2.9 ± 0.5 | 3.6 ± 0.5 | 4.9 | 1.3 ± 0.0 | 0.8 ± 0.1 | 1.2 ± 0.1 |
| 45 | 5.4 ± 0.8 | 2.1 ± 0.3 | 2.3 ± 0.4 | 3.7 ± 0.9 | 6.1 ± 1.0 | 9.4 | 1.6 ± 0.2 | 1.1 ± 0.1 | 0.9 ± 0.0 |
| 48 | 8.0 ± 0.7 | 2.0 ± 0.1 | 2.4 ± 0.2 | 4.6 ± 0.4 | 4.9 ± 0.4 | 5.6 | 1.3 ± 0.1 | 1.1 ± 0.1 | 1.5 ± 0.2 |
| 50 | 9.7 ± 1.7 | 2.6 ± 0.5 | 2.2 ± 0.5 | 4.8 ± 1.2 | 8.6 ± 0.7 | 7.2 | 3.0 ± 0.3 | 1.5 ± 0.1 | 1.0 ± 0.2 |
| *T_P_* (°C ) | 43.4 ± 0.9 | 38.4 ± 3.1 | N.A. | 40.6 ± 2.7 | N.A. | N.A. | N.A. | N.A. | N.A. |

*T_p_* in the temperature response of *g_min_* was determined by segmented linear regression analyses. N.A. indicates no significant braking points in segmented linear regression

**Table S2.** Summary of the linear regression models. T is temperature (numeric), Species is tree species tested in the study (categorical), and Sample indicates the leaf and shoot samples used in 2021 and 2022, respectively (categorical). SS is the sum of squares, DF is the degree of freedom. Significant p values are shown in bold.

| Model | Effects | SS | DF | F value | *P*-value |
| --- | --- | --- | --- | --- | --- |
| ln(*g_min_*) = T * Species * Sample | T | 7.49 | 1 | 316.07 | **<0.001** |
|  | Species | 27.93 | 8 | 147.25 | **<0.001** |
|  | Sample | 0.79 | 1 | 33.32 | **<0.001** |
|  | T : Species | 0.48 | 8 | 2.55 | **0.026** |
|  | T : Sample | 0.03 | 1 | 1.46 | 0.24 |
|  | Species : Sample | 0.65 | 8 | 3.42 | **0.005** |
|  | T : Species : Sample | 0.20 | 8 | 1.03 | 0.43 |

**Table S3.** Summary of linear regression between temperature, VPD and *g_min_* for 9 studied tree species (*Acer pseudoplatanus*, *Carpinus betulus*, *Fagus sylvatica*, *Fraxinus excelsior*, *Quercus sp*., *Sorbus torminalis*, *Abies alba*, *Picea abies*, *Pinus sylvestris*). *g_min_* was log transformed. R^2^ indicates the adjusted R-squared.

|  | Ln(*g_min_*) ~ Temperature | | Ln(*g_min_*) ~ VPD | |
| --- | --- | --- | --- | --- |
|  | R^2^ | *p* | R^2^ | *p* |
| *Acer* | 0.75 | **<0.01** | 0.22 | 0.14 |
| *Carpinus* | 0.80 | **<0.01** | 0.21 | 0.14 |
| *Fagus* | 0.89 | **<0.001** | 0.39 | 0.06 |
| *Fraxinus* | 0.81 | **<0.01** | 0.35 | 0.07 |
| *Quercus* | 0.60 | **<0.05** | 0.11 | 0.41 |
| *Sorbus* | 0.71 | **<0.01** | 0.11 | 0.22 |
| *Abies* | 0.66 | **<0.01** | 0.16 | 0.33 |
| *Picea* | 0.64 | **<0.01** | 0.12 | 0.40 |
| *Pinus* | 0.51 | **<0.05** | 0.93 | **<0.001** |
